# Pre-expression of a sulfhydryl oxidase significantly increases the yields of eukaryotic disulfide bond containing proteins expressed in the cytoplasm of *E.coli*

**DOI:** 10.1186/1475-2859-10-1

**Published:** 2011-01-07

**Authors:** Van Dat Nguyen, Feras Hatahet, Kirsi EH Salo, Eveliina Enlund, Chi Zhang, Lloyd W Ruddock

**Affiliations:** 1 Department of Biochemistry, Linnanmaa Campus, University of Oulu, 90570 Oulu, Finland

## Abstract

**Background:**

Disulfide bonds are one of the most common post-translational modifications found in proteins. The production of proteins that contain native disulfide bonds is challenging, especially on a large scale. Either the protein needs to be targeted to the endoplasmic reticulum in eukaryotes or to the prokaryotic periplasm. These compartments that are specialised for disulfide bond formation have an active catalyst for their formation, along with catalysts for isomerization to the native state. We have recently shown that it is possible to produce large amounts of prokaryotic disulfide bond containing proteins in the cytoplasm of wild-type bacteria such as *E. coli *by the introduction of catalysts for both of these processes.

**Results:**

Here we show that the introduction of Erv1p, a sulfhydryl oxidase and a disulfide isomerase allows the efficient formation of natively folded eukaryotic proteins with multiple disulfide bonds in the cytoplasm of *E. coli*. The production of disulfide bonded proteins was also aided by the use of an appropriate fusion protein to keep the folding intermediates soluble and by choice of media. By combining the pre-expression of a sulfhydryl oxidase and a disulfide isomerase with these other factors, high level expression of even complex disulfide bonded eukaryotic proteins is possible

**Conclusions:**

Our results show that the production of eukaryotic proteins with multiple disulfide bonds in the cytoplasm of *E. coli *is possible. The required exogenous components can be put onto a single plasmid vector allowing facile transfer between different prokaryotic strains. These results open up new avenues for the use of *E. coli *as a microbial cell factory.

## Background

Disulfide bonds are covalent linkages formed between two cysteine residues in proteins. Around one-third of all human proteins fold in the endoplasmic reticulum (ER) and acquire disulfide bonds there. The formation of native disulfide bonds is often the rate limiting step of protein biogenesis [1-3]. Due to this the production of proteins that contain disulfide bonds is difficult, especially on a large scale.

In compartments where disulfide bond formation naturally occurs there are two distinct steps in biosynthesis, catalysis of *de novo *disulfide bond formation and subsequent rearrangement or isomerization to generate native disulfide bonds. In order to produce disulfide bond containing proteins in the cytoplasm of *E. coli *a variety of strains have been produced [4-11]. These are available commercially under the names origami or rosetta-gami (Novagen) or SHuffle (New England Biolabs) and have the common feacture that they are Δ*gor *Δ*trxB *strains and hence have both of the disulfide bond reducing pathways in the cytoplasm disrupted. The SHuffle system also expresses DsbC in the cytoplasm as a catalyst of disulfide bond isomerization. While these systems generate disulfide bond containing proteins, the yields are often very low as there is no catalyst of *de novo *disulfide bond formation present.

Recently we showed that the introduction of a sulfhydryl oxidase, a natural catalyst of *de novo *disulfide bond formation, into the cytoplasm of *E. coli *allowed efficient production of disulfide bond containing prokaryotic proteins even without disruption of the reducing pathways [12]. In particular we used the sulfhydryl oxidase Erv1p from the inter-membrane mitochondrial space of *S. cerevisiae*.

Here we show that the introduction of Erv1p along with a catalyst of disulfide bond isomerization into the cytoplasm of wild-type *E. coli *results in efficient production of eukaryotic disulfide bond containing proteins. By combining the pre-expression of these two catalysts of native disulfide bond formation with methods to keep folding intermediates soluble and alternative growth media, expression levels of active complex proteins such as the nine disulfide bond containing vtPA from growth in shake flasks are over 800-fold greater than previously reported. These results open up new possibilities for the production of disulfide bond containing proteins in high yields in *E. coli*.

## Results

### Production of a tissue plasminogen activator fragment

Our previous systems using Erv1p as a catalyst of disulfide bond formation in the cytoplasm of *E. coli *were based on screening for an increase in yield of active *E. coli *alkaline phosphatase (PhoA; 2 disulfides) and *E. coli *Phytase (AppA; 4 disulfides). While these results showed that disruption of reducing pathways is not essential for efficient disulfide bond formation in the cytoplasm of *E. coli *and that high yields of active proteins were possible [12], we wanted to see if the use of Erv1p would allow for the production of more challenging proteins of eukaryotic origin.

**Figure 1:**
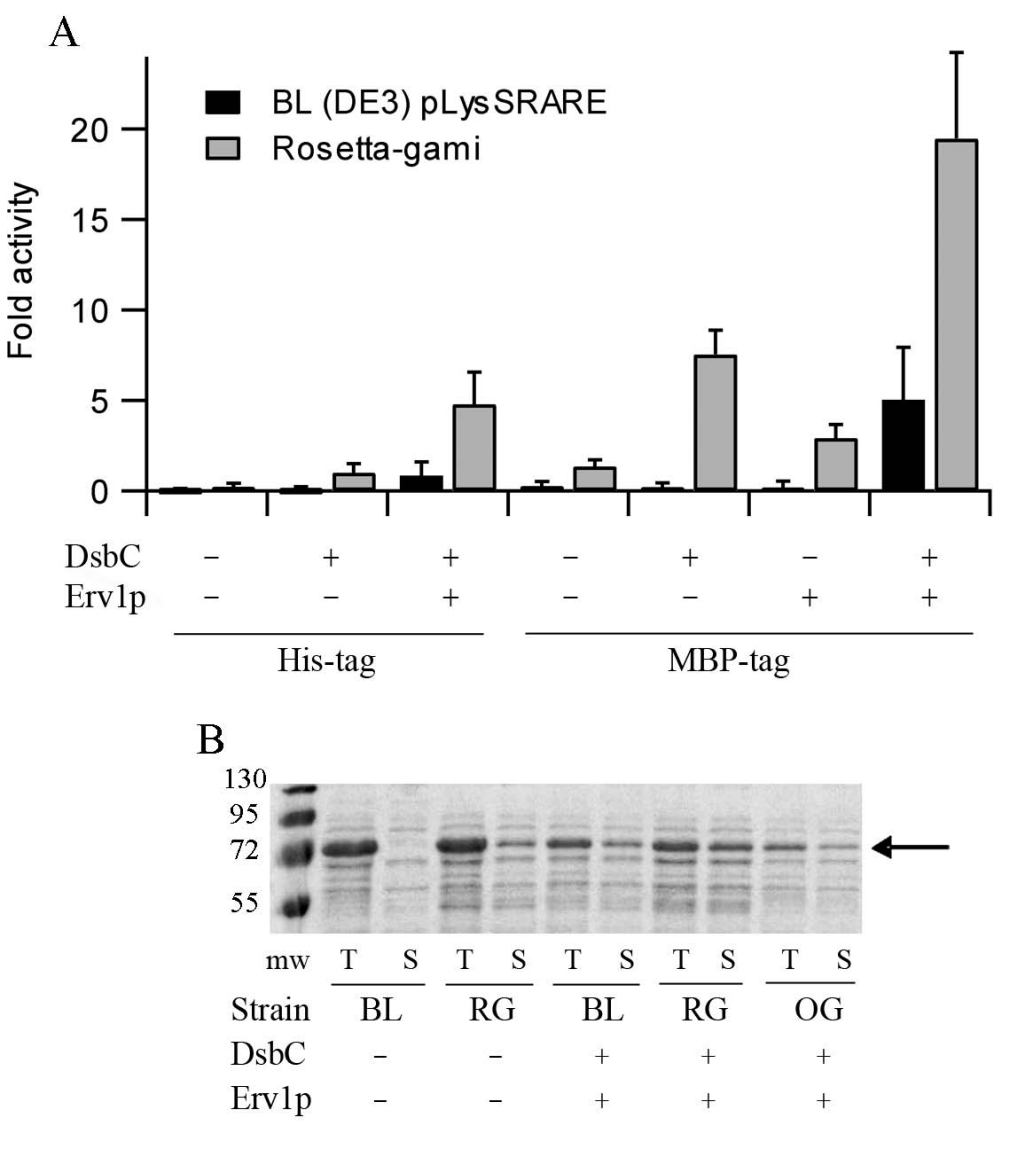
**Production of vtPA in the cytoplasm of *E. coli***. A) Relative yields of active vtPA produced in LB media at 30°C, normalized to the equivalent of the best previously reported system (a Δ*gor *Δ*trxB *strain with co-expression of mature DsbC) and shown as mean ± s.d. (n = 4**-**12; see additional file 1: Table of vtPA production and activity data). Strains: BL = BL21 (DE3) pLysSRARE; RG = rosetta-gami; OG = origami. When present Erv1p and mature DsbC are co-expressed from a polycistronic vector. B) SDS-PAGE analysis of MBP-fusion vtPA in LB media. T = total *E. coli *lysate, S = soluble fraction. The position of MBP-vtPA is marked with an arrow. Note the difference in expression levels in rosetta-gami compared with origami due to the expression of rare tRNAs.

Tissue plasminogen activator is a protease that converts plasminogen to plasmin and is used to treat pulmonary embolism, myocardial infarction and stroke. Retavase, one of the commercially available preparations, is a fragment of tissue plasminogen activator which contains only the kringle 2 and protease domains and as such it contains nine disulfide bonds. This fragment, also known as vtPA, has previously been used to characterize disulfide bond formation in the cytoplasm of *E. coli *[5]. Expression of vtPA in *E. coli *results in the formation of inactive inclusion bodies. While expression of vtPA in the Δ*gor *Δ*trxB *strain rosetta-gami results in a small gain of activity (Figure 1A and see additional file 1: Table of vtPA production and activity data), as per previously published results [5], the protein is still predominantly found in the insoluble fraction. Since vtPA contains a significant number of non-sequential disulfide bonds a disulfide isomerase is required for the more efficient formation of native disulfide bonds. The co-expression of the periplasmic disulfide isomerase DsbC in the cytoplasm of the Δ*gor *Δ*trxB *strain resulted in a significant increase in the amount of active protein produced (Figure 1A and see additional file 1: Table of vtPA production and activity data), as per previous results with vtPA derivatives [5]. While no significant activity could be seen for vtPA with or without co-expression of mature DsbC in a strain with the reducing pathways intact, co-expression of Erv1p and mature DsbC in this strain resulted in the production of active vtPA at comparable levels to that seen in the Δ*gor *Δ*trxB *strain with co-expression of mature DsbC alone (Figure 1A and see additional file 1: Table of vtPA production and activity data). Hence the disruption of genes involved in the reducing pathways in the cytoplasm is not obligatory for the production of even a complex disulfide bonded eukaryotic protein. However, in contrast to the results we previously observed for PhoA and AppA [12], the effects of co-expression of an active catalyst of disulfide bond formation were additive with disruption of the reducing pathways for vtPA (Figure 1A and see additional file 1: Table of vtPA production and activity data). While these results indicate that it is possible to increase the yield of active protein produced 5-fold over previously published results, we noted that a significant proportion of the vtPA produced was in mixed disulfide oligomers and hence not correctly folded (data not shown).

### Production of a tissue plasminogen activator fragment fusion protein

It is known that the production of proteins as fusion proteins can increase the solubility of the protein and hence increase the yield. This may be especially relevant to the production of disulfide bond containing proteins since the formation of the native state is slower, due to the requirement to form native disulfides, and hence the folding intermediates, which are usually considered to be more prone to aggregation than the native state, are longer lasting. We chose to use maltose binding protein (MBP) as a fusion partner since it contains no cysteine residues and hence cannot form additional non-native disulfide bonds and since it has been reported to be very efficient at increasing the yields of heterologously expressed proteins [13]. The expression of vtPA as a MBP fusion resulted in increased levels of active protein being produced when either Erv1p was co-expressed or the protein was expressed in the Δ*gor *Δ*trxB *strain, again with these effects being additive for vtPA (Figure 1A and see additional file 1: Table of vtPA production and activity data). Combining these, along with co-expression of a disulfide isomerase, resulted in activity levels circa 20-fold higher than that of co-expression of DsbC in a Δ*gor *Δ*trxB *strain and a concomitant increase in the yield of soluble protein (Figure 1B). While this is a very significant increase in active protein production, analysis of the vtPA produced suggested that a significant proportion of the material was still not correctly folded.

### Development of a pre-expression vector for Erv1p and a disulfide isomerase

**Figure 2:**
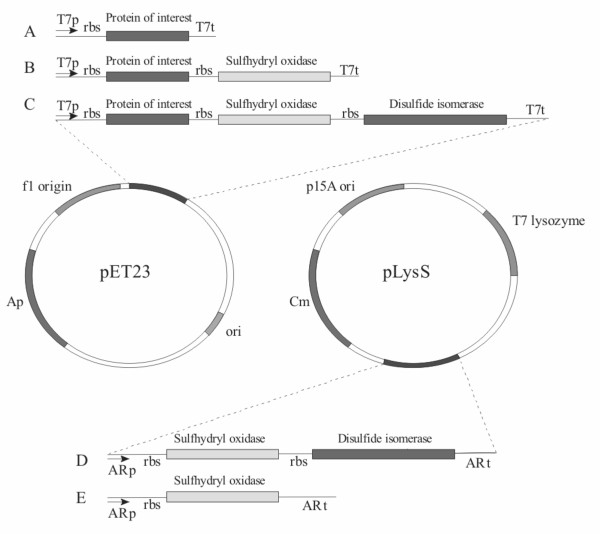
**Design of the plasmids for pre- and co-expression of Erv1p and/or a protein disulfide isomerase**. Co-expression is based on a polycistronic vector based on pET23 with a T7 promoter/terminator and IPTG induction. Pre-expression is based on cloning the Erv1p +/- the disulfide isomerase into an arabinose inducible system cloned into pLysS. Since the vector map for pLysSRARE is not available we were unable to clone into this vector and hence when using a Δ*gor *Δ*trxB *strain the co-expression studies are done in an origami rather than rosetta-gami background.

**Figure 3:**
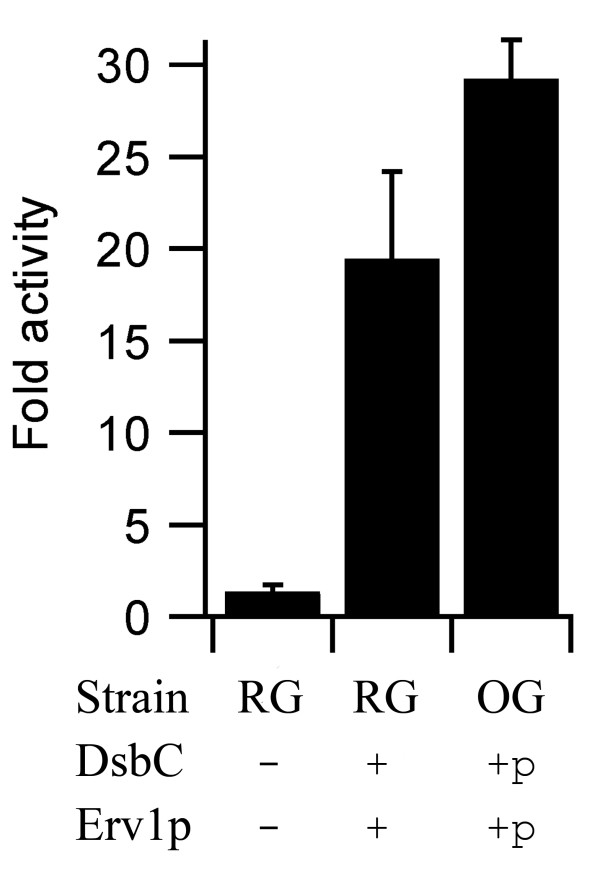
**The effect of co- vs pre-expression of folding factors on the production of vtPA in the cytoplasm of *E. coli***. Relative yields of active MBP-vtPA produced in LB media at 30°C, normalized to the equivalent of the best previously reported system (a Δ*gor *Δ*trxB *strain with co-expression of mature DsbC) and shown as mean ± s.d. (n = 4-12; see additional file 1: Table of vtPA production and activity data). Strains: RG = rosetta-gami; OG = origami. For Erv1p and mature DsbC expression + = co-expression while +p = pre-expression. Note the increase in vtPA activity with pre-expression comes despite the significant reduction in vtPA expression levels in rosetta-gami compared with origami due to the expression of rare tRNAs (figure 1B).

We thought that co-expression of the Erv1p and/or disulfide isomerase might be an issue for MBP-vtPA production. The first MBP-vtPA produced would experience low to non-existent levels of these folding factors and hence would not be able to fold correctly, and misfolding/aggregation is an auto-catalytic process. To circumvent this issue we made constructs which expressed either Erv1p alone or Erv1p and a disulfide isomerase from an arabinose promoter system (Figure 2). Such a system allows pre-expression of these folding factors. The use of such a two-step expression system for chaperone and folding catalyst production has previously been reported to beneficial for the production of soluble folded recombinant protein [14]. Due to a lack of available information on the plasmid pLysSRARE found in rosetta-gami strains we decided to use pLysS as the backbone for this new vector. Hence experiments undertaken with the pre-expression plasmid results in no over-expression of the rare tRNAs found on pLysSRARE. This has an effect on reducing the amount of vtPA produced ([15] and Figure 1B). Despite this effect, pre-expression of Erv1p (data not shown) or dual expression of Erv1p and DsbC resulted in a significant increase (p < 0.01; two-tailed T-test of two samples of unequal variance) in the amount of active vtPA produced compared to co-expression, with circa 30-fold higher levels of activity compared with the non-MBP tagged protein in rosetta-gami with co-expression of DsbC (Figure 3 and see additional file 1: Table of vtPA production and activity data, including direct comparison of pre- and co-expression in origami in EnBase media).

### Media effects on the yield of active vtPA

**Figure 4:**
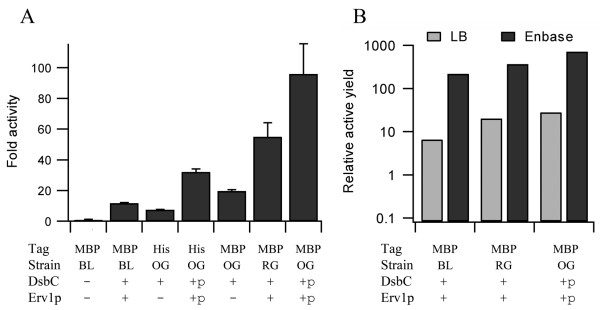
**Effects of media on vtPA production in the cytoplasm of *E. coli***. Panel A) Relative fold activity of vtPA produced in EnBase media at 30°C, normalized to the equivalent of the best previously reported system (rosetta-gami with co-expression of mature DsbC) and shown as mean ± s.d. (n = 2-6; see additional file 1: Table of vtPA production and activity data). Strains: BL = BL21 (DE3) pLysSRARE; RG = rosetta-gami; OG = origami. For Erv1p and mature DsbC expression + = co-expression while +p = pre-expression. Panel B) Relative yields of active vtPA made as an MBP fusion protein grown in LB and EnBase media in shake flasks at 30°C. Nomenclature as per the previous panel.

The production of proteins in prokaryotic systems is often very dependent on the media used. We therefore wanted to compare our expression system with that in the corresponding Δ*gor *Δ*trxB *strain in different media. Recently an ingenious media, marketed under the name EnBase (Biosilta Oy), has been reported which allows high density growth of *E. coli *cultures in shake-flasks via the controlled release of an utilizable carbon source [16]. The activity levels of vtPA produced in EnBase show similar overall patterns when compared to production in LB. However the relative activity per final OD_600 _of the culture was significantly increased in all comparisons made of vtPA expression (Figure 4A and see additional file 1: Table of vtPA production and activity data). The highest relative activity per OD_600 _of the final culture was MBP-tagged vtPA with pre-expression of Erv1p and DsbC which resulted in a circa 100-fold increase in relative activity c.f. the non-MBP tagged protein in rosetta-gami with co-expression of DsbC i.e. the best previously reported [7]. This increase in activity is possibly due to the slow bacterial growth in this media after induction [16] and hence the reduced rate of protein synthesis which decreases the probability of folding intermediates interacting with each other. In addition to this effect, the final optical density of cultures grown in shake flasks is circa 10-fold higher in EnBase media compared to the corresponding strains grown in LB media and hence the yields of active protein produced are up to 800-fold over the equivalent of the best previously reported in shake flasks (Figure 4B). While highly-active fully soluble monomeric MBP-vtPA can be produced in our system, the protein produced was still not homogenously correctly folded and after purification and cleavage of the MBP tag the vtPA had a tendency to aggregate making quantification and analysis of the protein produced difficult.

### Production of other eukaryotic proteins with multiple disulfide bonds

In addition to pre-expression of the folding factors being more efficient than co-expression, the synthesis of a pre-expression vector for Erv1p or Erv1p and a disulfide isomerase in theory obviates the need to make polycistronic vectors for their co-expression with target proteins, increasing their ease of use and facilitating transfer between different prokaryotic strains. To test this we examined the expression of a range of disulfide bond containing eukaryotic proteins in strains pre-expressing Erv1p and a disulfide isomerase from out pLysS-arabinose promoter based plasmids.

**Figure 5:**
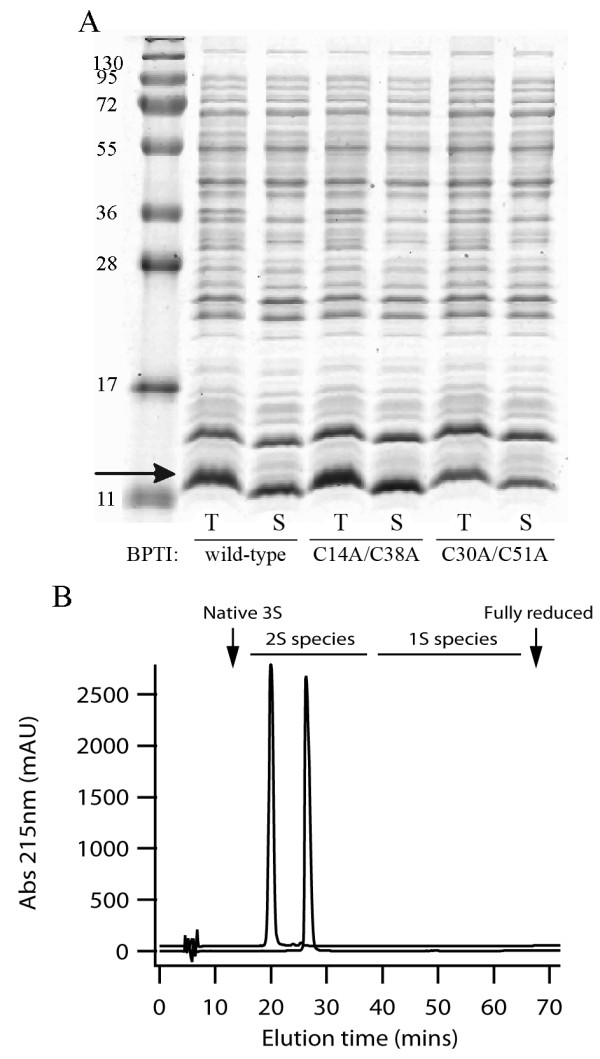
**Production of BPTI in the cytoplasm of *E. coli***. Analysis of mature BPTI wild-type and mutants which mimic the two disulfide folding intermediates produced in the cytoplasm of origami with pre-expression of Erv1p and PDI in LB media at 30°C. Panel A) Coomassie stained SDS-PAGE. T = total *E. coli *lysate; S = soluble fraction. The position of BPTI is marked with an arrow. Panel B) rpHPLC analysis of purified BPTI mutants under conditions which allow identification of the different folding intermediates. Upper trace (displaced 50 mAU) C30A/C51A mutant, lower trace C14A/C38A mutant. A single peak with the elution point being equivalent to the relevant two disulfide containing wild-type folding intermediate was obtained for both proteins.

Bovine pancreatic trypsin inhibitor (BPTI) is widely used as a model protein for examining disulfide bond formation *in vitro *[17-19]. The mature form is only 58 amino acids in size and contains three non-sequential disulfide bonds. We were interested in producing large amounts of mutants of BPTI that contain two disulfide bonds and mimic known folding intermediates for another project. In particular we wanted to produce a C14A/C38A mutant, which mimics the C5-C55/C30-C51 folding intermediate and the C30A/C51A mutant, which mimics the C5-C55/C14-C38 intermediate (note that the numbering used for BPTI follows the literature standard with numbering based on the mature protein sequence). Both species are thought to be major intermediates in the folding pathway [17] and contain two of the three disulfide bonds found in the native protein. When expressed in the cytoplasm of wild-type *E. coli *or the Δ*gor *Δ*trxB *strain rosetta-gami both mutants form inclusion bodies. In contrast when these mutants, or wild-type BPTI, are expressed in origami with pre-expression of Erv1p and mature PDI soluble protein is produced (Figure 5A). Purification and analysis of the C14A/C38A or C30A/C51A BPTI mutants revealed that both purified proteins were monomeric (data not shown), with their elution points from analytical reverse phase HPLC being equivalent to the relevant two disulfide containing wild-type folding intermediate (Figure 5B), indicating that only a single correctly folded disulfide bonded species is obtained. In contrast, when other mutants of BPTI were expressed that should mimic folding intermediates with one native and one non-native disulfide [18] e.g. C14A/C55A or C38A/C55A, no soluble protein was obtained under any conditions tested (data not shown). While fully soluble correctly folded BPTI mutants were produced upon pre-expression of Erv1p and mature PDI, pre-expression of Erv1p and mature DsbC under the same conditions resulted in a mixture of species, with only circa 50% of the protein correctly folded and the remainder forming inclusion bodies (data not shown). A similar beneficial effect of PDI expression on BPTI production in the periplasm of wild-type *E. coli*, where endogenous DsbC is present, has previously been reported [20].

**Figure 6:**
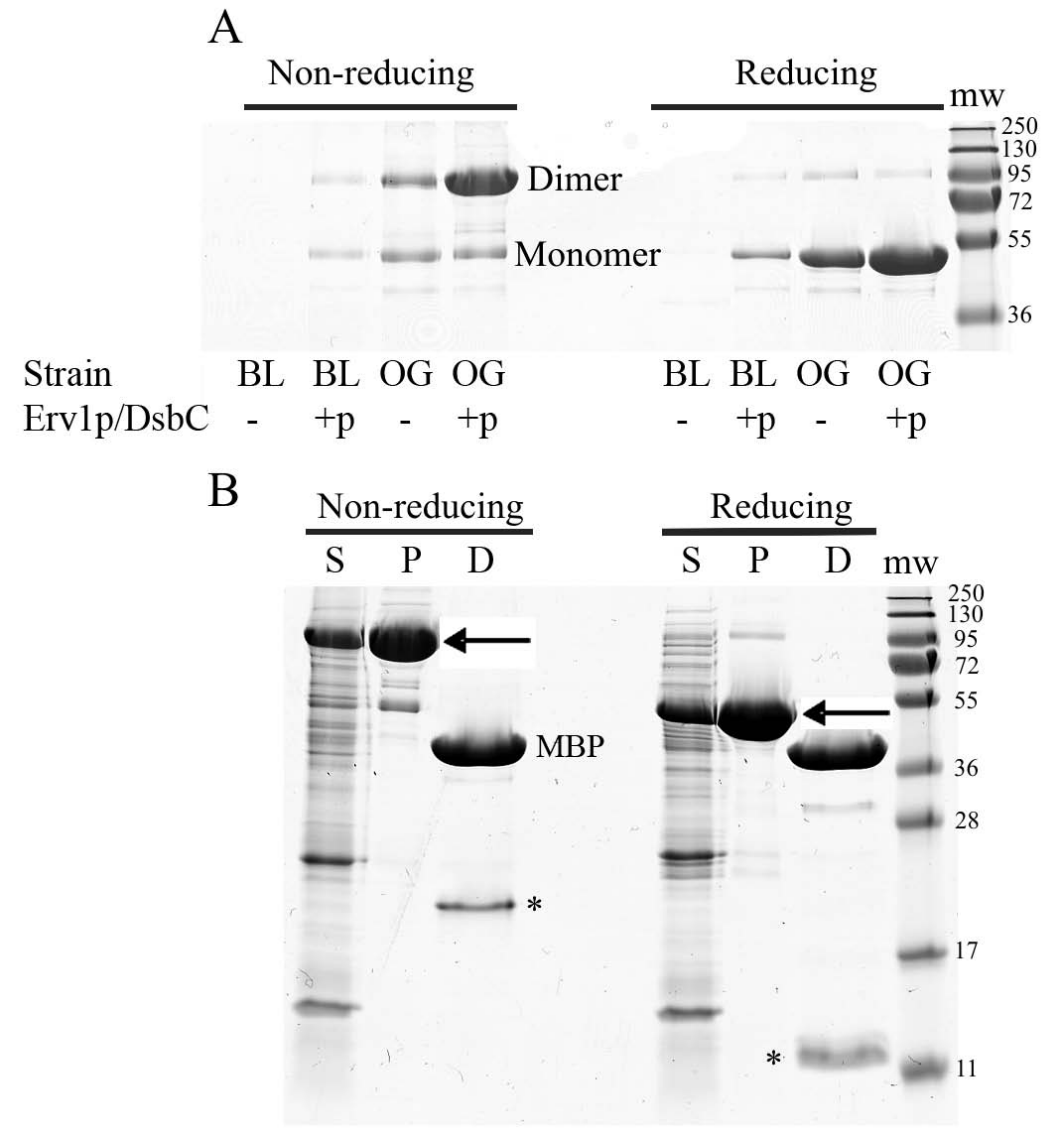
**Analysis of mature human resistin expressed in the cytoplasm of *E. coli *at 30°C in LB**. Panel A) Coomassie stained SDS-PAGE analysis of purified MBPx-resistin indicates production of the disulfide linked dimer increases with pre-expression of Erv1p and DsbC in both wild-type and Δ*gor *Δ*trxB *strains. The molecular weights (in kDa) of the markers (mw) are indicated as are the positions of the monomeric and dimeric forms of MBPx-resistin. Panel B) Coomassie stained SDS-PAGE analysis of mature resistin produced in origami with pre-expression of Erv1p and DsbC. S = soluble fraction of *E. coli *lysates; P = Purified MBPx-resistin from an amylose column; D = Purified material that has been digested with factor Xa to release MBP and resistin (marked with *). Note that under non-reducing conditions the resistin is a disulfide linked homo-dimer. The molecular weights (in kDa) of the markers (mw) are indicated as is the position of MBP post digestion. The position of the MBP-resistin fusion is indicated with an arrow.

All of the previous examples contain only intra-molecular disulfide bonds, but inter-molecular disulfide bond formation is important for many proteins. To examine whether our system is able to make inter-molecular disulfide bonded proteins we decided to examine production of resistin. Resistin is a hormone linked to the suppression of glucose uptake into adipose cells by insulin [21]. The mature protein is only 90 amino acids in size but it contains 5 non-sequential disulfide bonds and an inter-molecular disulfide which links two resistin monomers together to form a disulfide linked homodimer. Expression of MBP-tagged resistin with a factor Xa cleavage site in the linker region in BL21 (DE3) pLysS resulted in the production and purification of negligible soluble protein, while co-expression of Erv1p and DsbC resulted in the purification of some soluble protein (Figure 6A, compare lanes 1 and 2). Use of the origami strain further increased the yields of soluble protein produced but a heterogeneous population of species was obtained with the native dimeric state being in the minority. In contrast, expression in origami with pre-expression of Erv1p and mature DsbC resulted predominantly in the formation of resistin disulfide linked homodimers (Figure 6A compare lanes 3 and 4). Analysis of these species after purification using a one-step procedure amylose resin revealed that >90% of the material purified was a resistin homodimer (Figure 6B) and an Elmans assay revealed 0.02 free thiol groups per resistin molecule. Furthermore, cleavage of the mature resistin from the MBP using Factor Xa resulted in the production of MBP and a disulfide-linked homodimer of resistin (Figure 6B; note the relative size of MBP and resistin means that the relative staining is more than four times stronger for MBP for equivalent molar amounts of protein).

**Figure 7:**
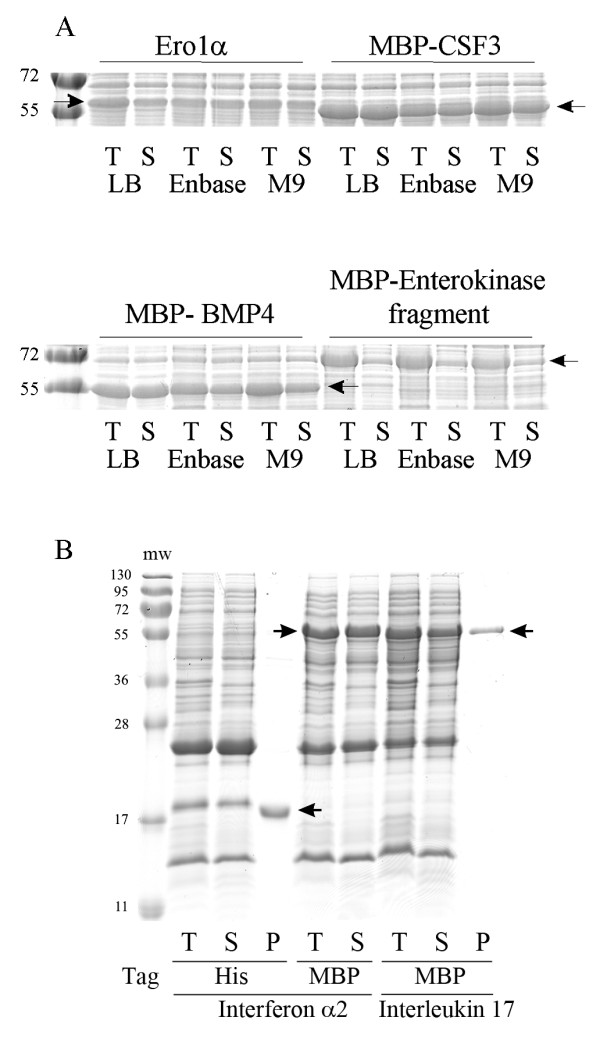
**Screening for the production of for other human proteins using Erv1p plus a disulfide isomerase pre-expression system**. Panel A) Coomassie stained reducing SDS-PAGE analysis of mature human proteins produced in the cytoplasm of BL21 (DE3) pLysS with different growth media. T = total *E. coli *lysate S = soluble fraction. The positions of the proteins are marked with arrows. The data shown is from Ero1α with pre-expression of Erv1p and mature PDI; MBP-tagged CSF3 with pre-expression of Erv1p and mature DsbC and co-expression of Erv1p and mature PDI; MBPx-tagged BMP4 with pre-and co-expression of Erv1p and DsbC; MBPx-light chain of enterokinase Cys896Ser with pre-expression of Erv1p and DsbC. Many other variants and combinations of pre- and co-expression conditions have been tested (see additional file 2: Table of vectors used in this study). For all four of these proteins under all conditions tested there is a concomitant increase in the number of disulfide bonds and yields of monomeric protein upon co- or pre-expression of Erv1p and a disulfide isomerase. Panel B) Reducing SDS-PAGE analysis of mature human proteins produced in the cytoplasm of origami in EnBase media with pre-expression of Erv1p and DsbC. T = total *E. coli *lysate, S = soluble fraction, P = protein purified using an NTA-spin column or amylose column. The molecular weights (in kDa) of the markers (mw) are indicated.

In addition to the more detailed studies on the model proteins PhoA [12], AppA [12], vtPA, BPTI mutants and resistin, we wanted to screen a wider range of human proteins to see if they could be made using the same Erv1p plus disulfide isomerase pre-expression vectors. These include production of:

i) Mature human Ero1α. The Ero1 family are ER-resident sulfhydryl oxidases involved in disulfide bond formation [22]. Human Ero1α contains multiple disulfide bonds which play a role in catalysis, regulation and some which are probably structural in nature [23,24]. It is produced insolubly in BL21(DE3) pLysSRARE and is predominantly insolubly produced in origami unless expressed as a fusion protein [24]. With pre-expression of Erv1p and human PDI it is expressed solubly in a range of media (Figure 7A)

ii) Mature human colony stimulating factor 3. CFS3 is a hormone that stimulates the production of granulocytes and stem cells [25] and is also known as granulocyte colony-stimulating factor. It is used in oncological and hematological applications. CSF3 contains two sequential disulfide bonds and one free thiol group near the N-terminal of the protein. With pre-expression of Erv1p and DsbC, when expressed as an MBP-fusion protein it is expressed solubly in a range of media (Figure 7A).

iii) Mature human bone morphogenic protein 4. BMP4 is a cytokine that regulates the formation of teeth, limbs and bones it plays a role in fracture repair and it initiates human stem cell differentiation to trophoblasts [26]. It contains three non-sequential intra-molecular disulfide bonds along with one inter-molecular disulfide bond. With pre-expression of Erv1p and DsbC, when expressed as an MBP-fusion protein it is expressed solubly in a range of media (Figure 7A).

iv) The catalytic light chain of human enterokinase. Enterokinase is a highly specific protease [27], used academically to release fusion proteins and to remove FLAG-tags from proteins. The catalytic light chain, expressed here, is 235 amino acids in size and contains four disulfide bonds plus a free thiol group that forms an intra-chain disulfide bond in the full length protein. With pre-expression of Erv1p and DsbC, when expressed as an MBP-fusion protein it is expressed in a partially soluble form in a range of media (Figure 7A).

v) Mature human interferon α2. Interferon α2 is an anti-viral protein produced by macrophages [28]. The mature human protein is 165 amino acids in length and it contains four cysteine residues that form two non-sequential disulfide bonds. When expressed with or without a MBP-tag with pre-expression of Erv1p and DsbC it is expressed solubly and can be purified to homogeneity (Figure 7B).

vi) Mature human interleukin 17. Interleukin 17 is a cytokine that induces the production of proinflamatory cytokines [29]. The mature human protein is 132 amino acids in length. It contains six cysteines which are reported to form two non-sequential intra-molecular disulfide bonds. When expressed with a MBP-tag with pre-expression of Erv1p and DsbC it is expressed solubly and can be purified to homogeneity (Figure 7B).

While these have not all been fully characterized in this system, the tests we have performed have all been positive, resulting in increased soluble yields of these disulfide bond containing proteins.

## Discussion

Currently it is often difficult to produce disulfide bond containing proteins, especially on a large scale. This severely inhibits scientific progress in understanding a myriad of mechanistic processes and imposes limitations on the biotechnology industry for the production of therapeutic proteins. The most common route for large scale production of proteins that contain disulfide bonds involves biosynthesis in the cytoplasm of *E. coli*. Here there are no mechanisms for disulfide bond formation. Due to this recombinant proteins are unable to attain their native conformation and form insoluble inclusion bodies. Inclusion body refolding is a widely studied field. However, it can be costly, complex, time consuming and is often inefficient. Alternative routes for producing disulfide bonded proteins such as periplasmic expression or expression in the endoplasmic reticulum of eukaryotes also have drawbacks.

Current systems for making disulfide bonded proteins in the cytoplasm of *E. coli *are based on the disruption of the thioredoxin/thioredoxin reductase and glutathione/glutathione reductase pathways present in this cytoplasm [4-11]. We recently showed that by co-expression of a catalyst of disulfide bond formation prokaryotic disulfide bond containing proteins could be produced even without disruption of these reducing pathways [12]. Here we further develop and test this system for the production of eukaryotic proteins with multiple disulfide bonds.

For proteins with only a few disulfide bonds e.g. PhoA and AppA, we showed that the addition of a catalytic system for the formation of disulfide bonds i.e. co-expression of the sulfhydryl oxidase Erv1p, increased the yields of active protein produced up to 1000-fold over the wild-type *E. coli *control, with yields of up to 16 mg/L of culture [12]. Furthermore, the addition of a catalyst for the formation of disulfide bonds is more effective than the removal of the reducing pathways for these proteins i.e. BL21 (DE3) pLysSRARE with co-expression of Erv1p generates significantly higher yields of active disulfide bond containing PhoA or AppA than the rosetta-gami Δ*gor *Δ*trxB *strain [12]. However, here we show for proteins with more disulfide bonds e.g. vtPA, we find consistently that production via co- or pre-expression of Erv1p in a Δ*gor *Δ*trxB *strain background is more efficient than in a wild-type background, presumably due to the lack of competition of the reverse reduction pathway. For all proteins we have tested that are able to show at least some folding to the native state in *E. coli*, co-expression of Erv1p, plus a disulfide isomerase where needed, significantly increases the yield of active and/or solubly produced protein independent of the genetic background of the strain. In addition, the system is not limited to the use of Erv1p, other sulfhydryl oxidases can potentially be used. For example, we have preliminary data showing that co-expression of human Ero1α and PDI is able to catalyze folding of vtPA in a Δ*gor *Δ*trxB *strain (Ero1α itself contains structural disulfides and active protein is not obtained in a wild-type background, though soluble protein can be made in BL21 through the pre-expression of Erv1p and PDI; Figure 7A)

The use of a fusion partner e.g. MBP, to increase the solubility of the folding intermediates also contributes significantly to increasing the yields of disulfide bonded proteins as does pre-expression of the folding factors and the choice of media. In combination we are able to get yields of active vtPA circa 800 fold higher than previously reported using DsbC co-expression in a Δ*gor *Δ*trxB *strain. However, the system as reported here is not perfect. For proteins with only a few disulfide bonds the material can be produced in high yields and can be homogenous, especially when a fusion partner is used. However, resistin (with 5 disulfide bonds plus an inter-molecular disulfide) is the most complex protein we have been able to produce to near homogeneity to date. More complex proteins e.g. vtPA show greatly increased levels of activity and/or disulfide bond formation but homogeneous natively folded protein is not observed. However, it is clear that optimization of the system in terms of timing, levels and nature of the pre-expressed folding factors e.g. which disulfide isomerase is used and growth conditions can make significant differences to the quality of the protein obtained and that this needs to be optimized for individual proteins rather than the use of standard expression conditions for all proteins reported here.

## Conclusions

By using the components of natural systems and expressing a catalyst of disulfide bond formation and a catalyst of disulfide bond isomerization we are able to establish systems which allow for the efficient production of eukaryotic proteins with multiple disulfide bonds in the cytoplasm of *E. coli*. While the system still requires future development, we are able to get yields of active vtPA circa 800 fold higher than previously reported using DsbC co expression in a Δ*gor *Δ*trxB *strain. Despite the requirement for individual optimization for more complex proteins our results opens up a wide range of possibilities for the academic investigation of structure/function relationships of a large body of proteins whose production was previously a limitation and potentially the production of disulfide bonded proteins on an industrial scale.

## Methods

### Vector construction

Expression vectors (see additional file 2: Table of vectors used in this study) were made by standard molecular biology techniques.

Expression vectors for Erv1p, mature DsbC, mature PhoA, mature AppA, mature PDI and mature BPTI were constructed previously [12,30,31]. The gene for mature MBP (Lys27-Thr392) was amplified by PCR using a colony of *E. coli *XL1-Blue as a template. The genes for the kringle 2 and protease domains of human tissue plasminogen activator (vtPA; Gly211-Pro562), mature human resistin (Lys19-Pro108), mature human CSF3 (Ala30-Pro207), mature human BMP4 (Pro294-Arg408), mature human Ero1α (Glu24-His468), human enterokinase light chain (Ile785-His1019), mature human interferon α2 (Cys24-Glu188) and mature human interleukin 17 (Gly24-Ala155) were amplified using IMAGE clones as templates.

Mature BPTI, human vtPA, human tissue factor luminal domain, human CSF3, human Ero1α, human enterokinase light chain and human interferon α2 were cloned into a modified version of pET23a which includes an N-terminal his-tag in frame with the cloned gene and an additional SpeI site in the multi-cloning site between the EcoRI and SacI sites. The resulting gene products include the sequence MHHHHHHM- prior to the first amino acid of the protein sequence.

Polycistronic vectors were constructed by taking fragments encoding the folding factors from the pET23 based constructs which include the ribosome binding site e.g. XbaI/X fragments and ligating them into the SpeI/X cut plasmid encoding the protein of interest (where X is an appropriate restriction site found in the multi-cloning site after SpeI and not found in either gene e.g. XhoI). After a single such ligation this generates a plasmid that contains a single transcription initiator/terminator and hence makes a single mRNA, but has two ribosome binding sites and makes two proteins by co-expression from two translation initiation sites (Figure 2A). This ligation results in the loss of the original SpeI site. Transfer of a SpeI site after the second gene into the new vector allows a third gene to be cloned by the same method resulting in a tricistronic vector which makes three proteins from a single mRNA. Note that to clone mature human PDI into these polycistronic vectors silent mutations were made in the two internal XhoI sites in the gene.

A variety of MBP-fusion protein constructs were made. All were based on the expression of mature MBP (Lys27-Thr392) cloned NcoI/BamHI into pET23 d. At the 3' end of the MBP gene a variety of flexible linkers were introduced. These were: i) NSSSNNNNHM; ii) GSGSGSGSGSIEGRGSGSGSGSGSHM, allowing cleavage by Factor Xa and iii) GSGSGSGSGSDDDDKHM, allowing cleavage by enterokinase, with all three having a NdeI restriction site encoded by the terminal His-Met to allow construction of the fusion protein. Several of the proteins of interest were tested in two or more of these MBP variants and for most no significant differences were observed between the variants. vtPA was tested in all three variants and the activity obtained with the first variant was significantly lower (circa 30%) than for the other two under all conditions tested. Due to the relative costs of the proteases the Factor Xa containing linker version of the MBP-fusion protein construct was our version of choice here (denoted MBPx) and all fusion protein data shown here is that vector unless otherwise stated.

The generation of the pre-expression vectors was complicated due to the lack of suitable restriction sites between our chosen host vector (pLysS), the pET23 based vectors we had already cloned our folding factors into and the pBAD 102/D-TOPO vector (Invitrogen) from which we cloned the arabinose promoter. pLysS was chosen as the backbone for the pre-expression vector as it, or a derivative of it is already in all of our host strains and it is fully compatible with co-transfection with many commercial expression vectors, not only pET23. We would also have liked to clone this system into pLysSRARE, but lack of available information on this vector hampered us. pLysS was modified with an NsiI site added at position 3071 and an AvrII site added at position 3578. pBAD 102/D-TOPO was modified by adding an AvrII site at 1089 a XbaI site at 316 and a XhoI site at 796. The genes encoding for Erv1p or Erv1p and mature DsbC or Erv1p and mature PDI were digested XbaI/XhoI from a pET23 based plasmid and cloned into the same sites in the modified pBAD102/D-TOPO. Note that this takes the ribosome binding sites from pET23 and removes a fragment from pBAD102/D-TOPO that includes the ribosome binding site, his-patch thioredoxin, enterokinase site, TOPO sites and V5 epitope. The genes of interest were then cloned NsiI/AvrII from this vector into the modified pLysS. This results in a modified pLysS that includes not only the genes of interest under the pBAD arabinose promoter, but also the *araC *gene for regulation.

Mutagenesis of plasmids was carried out using the QuikChange site-directed mutagenesis kit (Stratagene) according to the manufacturers' instructions.

All plasmid purification was performed using the QIAprep spin miniprep kit (Qiagen) and all purification from agarose gels was performed using the Gel extraction kit (Qiagen), both according to the manufacturers' instructions.

All plasmids generated were sequenced to ensure there were no errors in the cloned genes (see additional file 2: Table of vectors used in this study for plasmid names and details).

### Protein expression

For expression in LB media, *E. coli *strains containing expression vectors were streaked out from glycerol stocks stored at -70°C onto LB agar plates containing suitable antibiotics to allow for selection (100 μg/ml ampicillin for pET23 derivatives, 35 μg/ml chloramphenicol for pLysS derivatives; with 10 μg/ml tetracycline and 15 μg/ml kanamycin for selection of origami or rosetta-gami strains). The next day one colony from these plates were used to inoculate 5 ml of LB media, containing suitable antibiotics, and grown overnight at 30°C, 200 rpm. This overnight culture was used to seed a 50 ml culture of LB containing suitable antibiotics in a 250 ml conical flask to an optical density of 0.05 at 600 nm (OD600). The addition of FAD to the media is not required for the production of active Erv1p and preliminary experiments suggestion such addition does not increase the yield of active proteins generated in this system. This culture was grown at 30°C, 200 rpm until the OD600 reached 0.4 at which point protein production was induced either by the addition of 0.5 mM IPTG or for pre-induction of the folding factors by the addition of 0.5% w/v arabinose followed 30 minutes later by 0.5 mM IPTG. The cells were then grown for a total of 4 hours post induction at 30°C, 200 rpm and the final OD600 measured. The cells were collected by centrifugation and resuspended to an OD600 equivalent of 10 (based on the final OD_600 _of the culture) in 20 mM sodium phosphate pH 7.4, 20 μg/ml DNase, 0.1 mg/ml egg white lysozyme and frozen. Such normalization allows for easy correction for differences in the growth rates of the cultures and allows rapid equal total protein loading of samples for activity assay and SDS-PAGE analysis. Cells were lysed by freeze-thawing. Where appropriate, the insoluble fraction was removed by centrifugation and the soluble fraction removed quickly to a new container. Cell lysates or soluble fractions were stored frozen in 1 ml aliquots for further experiments as repeated freeze-thawing clearly influenced the results obtained in a protein dependent manner.

For expression in EnBase media (Biosilta Oy, Finland) *E. coli *strains containing expression vectors were streaked out from glycerol stocks stored at -70°C onto LB agar plates containing suitable antibiotics to allow for selection. The next day three loopfuls of bacteria from the region of single colonies were collected and resuspended in 0.8 ml of EnBase media. This was used to inoculate 25 ml of EnBase media (with thiamine, magnesium and Enz I'm pre-added according to the manufacturer's instructions), containing suitable antibiotics in a 250 ml conical flask to an OD600 of 0.1. The flask was then sealed with an oxygen permeable seal and the bacteria grown for 15 hours at 30°C, 200 rpm. The OD600 of the culture was measured to ensure correct growth and the booster media added according to the manufacturer's instructions. Pre-induction of the folding factors was performed by the addition of 0.5% w/v arabinose, the cells grown for 30 minutes and then the protein of interest induced with 0.5 mM IPTG and the cultures grown for a further 23.5 hours and the final OD600 measured. The post culture processing was as per growth in LB media.

Note, in addition to the results shown here many of the proteins of interest were also expressed under conditions other than those listed above. Varying the conditions altered the yields of active protein obtained, sometimes in a protein dependent manner. However, all of the results obtained from all conditions are consistent with the results reported here. While these consensus conditions may not be optimal for any individual protein in our system, they appear to be a good starting point for screening for any protein in our system. Note also that 30°C for growth was chosen as Erv1p is not well expressed in an active form at 37°C and that growth of large numbers of cultures was more convenient for us at 30°C than at lower temperatures. Some proteins, for example the luminal domain of tissue factor (data not shown), are expressed more homogeneously at lower temperatures, but the effect of temperature, induction OD_600_, induction time or the concentration of inducing agent has not been systematically tested for any protein.

### vtPA activity measurements from lysates

vtPA activity was measured using a chromagenic substrate, chromozyme t-PA peptide assay (Roche), using a methodology similar that that recommended by the manufacturer but adapted for a continuous assay in 96-well plate format. Since this method showed slight variations in activity with time all of the vtPA activity measurements were repeated using the same batch of substrate and same buffers and that data is presented here. 20 mg of substrate was dissolved in 4 ml of sterilized water to generate a 20× substrate stock solution. 10 μl of soluble fraction from cell lysates were added to 190 μl of substrate diluted in reaction buffer (100 mM tris-HCl, 0.15% tween20; pH 8.5) to give a final concentration that is 1× in a 96 well microtitre plate, thermally equilibrated to 37°C. The absorbance at 405 nm was measured at 3 minute intervals for 30 minutes to determine the rate of formation of the product. Background rates from appropriate controls strains not expressing vtPA were subtracted, with these being less than 5% of the signals for any system where Erv1p and a disulfide isomerase were co- or pre-expressed. For samples with little vtPA activity 20 μl of soluble fraction from cell lysates were added to 180 μl of 1× substrate (see additional file 1: Table of vtPA production and activity data). Control samples tested with 10 and 20 μl of lysate showed no significant differences in activity once normalized for the volume of lysate used. It should be noted that this assay format gives a very inaccurate measure for the production of vtPA with no co- or pre-expression of vtPA in a wild-type *E. coli *background. Furthermore, our vtPA constructs have different tags than previously reported and our activity measurements are made from *E. coli *lysates, both of which may affect activity measurements and hence cross-comparisons to available specific activity values are inappropriate. Hence all of the data has been normalized to that produced with co-expression of DsbC in a Δ*gor *Δ*trxB *background i.e. the best system previously reported. More accurate determinations of very low activities can be obtained under different experimental conditions (more lysate and much longer incubations), but Erv1p co- or pre-expression strains cannot be assayed under these conditions as the measurements go off scale. All samples were measured at least in duplicate. The number of samples (n) in Figures 1, 3 and 4 and Table 1 represent independent bacterial cultures, each measured at least in duplicate, implying the number of activity measurements for each data point varied from 4 to 24.

### Protein purification and analysis

Purification of hexa-histidine tagged proteins was performed by standard immobilized metal affinity chromatography using Ni-NTA columns (Qiagen) under native conditions was performed according to the manufacturers' instructions following clearance of the cell lysate by centrifugation (10000 rpm, 15 minutes, 4°C).

Immobilized metal affinity chromatography of N-terminal hexa-histidine tagged BPTI wild-type and mutants was performed using a 5 ml HiTrap Chelating HP column (GE healthcare). The soluble fraction of the *E. coli *lysate was prepared by centrifugation (10000 rpm, 15 minutes, 4°C). This was then loaded onto the HiTrap Chelating column which had been pre-charged with Ni^2+^, washed and equilibrated with 3 column volumes of 20 mM sodium phosphate (pH 7.4). After loading the sample the column was washed with 3 column volumes of wash buffer (20 mM sodium phosphate, 50 mM imidazole, 0.5 M sodium chloride; pH 7.4) then 3 column volumes of 20 mM sodium phosphate (pH 7.4) before elution with 2 column volumes of 50 mM EDTA (pH 7.4). All buffers were filtered and degassed before use.

Reverse phase high pressure liquid chromatography (rpHPLC) analysis of BPTI wild type and mutants produced in our strains was performed on an ÄKTA FPLC system (GE Healthcare) using a μRPC C2/C18 ST 4.6/100 column: The column was pre-equilibrated in buffer A (0.065% trifluoroacetic acid), before a 50 μl sample was loaded to the system using automatic sample injection. The elution gradients used were: 20-30% buffer B (90% acetonitrile, 0.05% trifluoracetic acid) over 3 column volumes followed by a column volume of 30% buffer B, then 30-31% buffer B over 5 column volumes, 31-45% buffer B over 5 columns volumes, 1 column volume of 45% buffer B for 1 column volume and then 45-100% buffer B over 3 column volumes. 0.5 ml fractions were collected with a flow rate of 0.3 ml/min. All buffers were filtered and degassed before use.

Purification of MBP-tagged proteins was performed using amylose resin (New England Biolabs). Amylose resin (500 μl for 5 ml of lysate) was packed into an empty plastic column. The resin was washed twice with 2 ml of water and then equilibrated with 5 ml of 20 mM sodium phosphate buffer (pH 7.4). The soluble fraction of the *E. coli *lysate was loaded on the column and allowed to equilibrate at room temperature for 5 minutes. The column was then washed five times with 2 ml of wash buffer (20 mM sodium phosphate, 0.2 M sodium chloride pH 7.4). 150 μl of elution buffer (20 mM sodium phosphate, 10 mM maltose, 0.2 M sodium chloride, pH 7.4) was added and the column allowed to equilibrate at room temperature for 5 minutes. The elution was repeated two more times.

For Factor Xa cleavage 5 μl of 10× Xa-buffer (New England Biolabs) was added to 43 μl of protein solution (in the MBP-purification elution buffer) with a final amount of protein of circa 50 μg. 2 μl of 1 mg/ml Factor Xa (New England Biolabs) was added, mixed and the solution incubated for 6 hours at room temperature. The soluble and insoluble fractions were subsequently analysed by reducing and non-reducing SDS-PAGE.

Elman's assay for free thiol content were performed at room temperature under denaturing conditions in 50 mM Tris buffer, 2 M Guanidine hydrochloride (pH 8.0) using 0.073 mg/ml Elman's reagent. The change in absorbance at 412 nm was monitored after 15 minutes and the free thiol content calculated using a molar extinction coefficient of 13600 M^-1^cm^-1^.

## Competing interests

A patent application has been filed.

## Authors' contributions

VDN and FH participated in the design of the research. VDN, FH, KEHS, EE and CZ performed the research. LWR conceived and coordinated the study, participated in its design, performed the research and wrote the manuscript. All authors read and approved the final manuscript.

## Supplementary Material

Additional file 1 **Table of vtPA production and activity data**. Details the vtPA activity data used to generate Figures 1, 3 and 4, including sample details, number of samples for each expression condition, lysate volume assayed, Δabsorbance values from the activity data and relative activity. Click here for file

Additional file 2 **Table of vectors used in this study**. Details the 59 plasmid vectors used in this study including plasmid name, the plasmid backbone and details of the protein(s) produced. Click here for file
