# Supplementary material for: Pre-expression of a sulfhydryl oxidase significantly increases the yields of eukaryotic disulfide bond containing proteins expressed in the cytoplasm of E.coli
Source: Microb Cell Fact. 2011 Jan 7;10:1. doi: 10.1186/1475-2859-10-1 (PMC3022669; doi:10.1186/1475-2859-10-1)
Supplement: Additional file 1 — Table of vtPA production and activity data. Details the vtPA activity data used to generate Figures 1, 3 and 4, including sample details, number of samples for each expression condition, lysate volume assayed, Δabsorbance values from the activity data and relative activity. [file 1475-2859-10-1-S1.PDF]

Additional file 1: Table of vtPA production and activity data

Strain	Tag	Co-expression	Pre-expression	Media	Lysate vol (μl)	n	corrected Δ Absorbance	Relative activity
BL21 (DE3)	His	-	-	LB	20	4	0.022 ± 0.094	0.05 ± 0.21
BL21 (DE3)	His	DsbC	-	LB	20	4	0.032 ± 0.139	0.07 ± 0.31
BL21 (DE3)	His	Erv1p and DsbC	-	LB	20	4	0.545 ± 0.395	1.21 ± 0.88
Rosetta-gami	His	-	-	LB	20	4	0.106 ± 0.083	0.24 ± 0.18
Rosetta-gami	His	DsbC	-	LB	20	4	0.449 ± 0.222	1.00 ± 0.49
Rosetta-gami	His	Erv1p and DsbC	-	LB	20	4	2.16 ± 0.79	4.80 ± 1.75
BL21 (DE3)	MBPx	-	-	LB	20	4	0.200 ± 0.116	0.44 ± 0.26
BL21 (DE3)	MBPx	DsbC	-	LB	20	4	0.198 ± 0.126	0.44 ± 0.28
BL21 (DE3)	MBPx	Erv1p	-	LB	20	4	0.140 ± 0.157	0.31 ± 0.28
BL21 (DE3)	MBPx	Erv1p and DsbC	-	LB	20	9	2.53 ± 1.39	5.63 ± 3.09
Rosetta-gami	MBPx	-	-	LB	20 or 10	4	0.610 ± 0.159 *	1.36 ± 0.36 *
Rosetta-gami	MBPx	DsbC	-	LB	20	4	3.39 ± 0.60	7.53 ± 1.34
Rosetta-gami	MBPx	Erv1p	-	LB	20	4	1.31 ± 0.34	2.92 ± 0.76
Rosetta-gami	MBPx	Erv1p and DsbC	-	LB	20 or 10	12	8.76 ± 2.11 *	19.5 ± 4.7 *
Origami	MBPx	-	Erv1p and DsbC	LB	10	6	6.57 ± 0.48	29.2 ± 2.1
Origami	MBPx	Erv1p and DsbC	Erv1p and DsbC	LB	10	4	7.85 ± 2.71	34.9 ± 12.0
Origami	His	DsbC	-	EnBase	10	2	1.70 ± 0.05	7.54 ± 0.22
Origami	His	-	Erv1p and DsbC	EnBase	10	2	7.21 ± 0.43	32.1 ± 1.9
BL21 (DE3)	MBPx	-	-	EnBase	10	2	0.164 ± 0.130	0.73 ± 0.58
BL21 (DE3)	MBPx	Erv1p and DsbC		EnBase	10	2	2.87 ± 0.10	12.8 ± 0.2
Origami	MBPx	-	Erv1p and DsbC	EnBase	10	6	21.6 ± 4.4	96.0 ± 19.6
Origami	MBPx	Erv1p and DsbC	-	EnBase	10	4	10.1 ± 1.8	44.9 ± 8.0
Origami	MBPx	DsbC	-	EnBase	10	2	4.42 ± 0.19	19.7 ± 0.8
Rosetta-gami	MBPx	Erv1p and DsbC	-	EnBase	10	2	12.3 ± 2.0	55.0 ± 9.2

Data for the production of vtPA grown in shake flasks at 30°C, where Δ absorbance is the rate of change in absorbance per minute (mAU/min) at 405 nm upon assaying the lysate, with each sample being measured at least in duplicate. The relative activity is calculated

relative to the mean value obtained for H_6-vtPA in rosetta-gami with co-expression of DsbC. Note that both the BL21 strain and the rosetta-gami (Δ*gor* Δ*trxB*) strain contain pLysSRARE, while the origami strain contains pLysS. The expression of DsbC, PDI and/or Erv1p did not result in significant changes to the background activity. However, there were significant differences in the background activity of different *E.coli* strains. * For the samples assayed using two different volumes of lysate there were no significant differences between the relative activities obtained and the corrected absorbances shown are relative to 20 μl of lysate.
