# Supplementary material for: Pre-expression of a sulfhydryl oxidase significantly increases the yields of eukaryotic disulfide bond containing proteins expressed in the cytoplasm of E.coli
Source: Microb Cell Fact. 2011 Jan 7;10:1. doi: 10.1186/1475-2859-10-1 (PMC3022669; doi:10.1186/1475-2859-10-1)
Supplement: Additional file 2 — Table of vectors used in this study. Details the 59 plasmid vectors used in this study including plasmid name, the plasmid backbone and details of the protein(s) produced. [file 1475-2859-10-1-S2.PDF]

Additional file 2: Table of vectors used in this study.

Plasmid	Basis	Protein being produced	Co-expression
pOLR130	pET23	Mature human PDI (Asp18-Leu508) with silent mutations to remove internal XhoI sites	-
pFH198	pET23	Mature *E.coli* DsbC (Asp21-Lys236)	-
pVD157	pET23	*S.cerevisiae* Erv1p (Met 1-Glu189)	-
pVD158	pET23	*S.cerevisiae* Erv1p (Met 1-Glu189)	DsbC
pVD159	pET23	*S.cerevisiae* Erv1p (Met 1-Glu189)	PDI
pJKJ16	pET23	Mature *E.coli* MBP (Lys27-Thr392) plus a linker NSSSNNNNHM	-
pVD110	pET23	Mature *E.coli* MBP (Lys27-Thr392) plus a linker GSGSGSGSGSIEGRGSGSGSGSGSHM (allowing Factor Xa cleavage)	-
pVD118	pET23	Mature *E.coli* MBP (Lys27-Thr392) plus a linker GSGSGSGSGSDDDDKHM (allowing enterokinase cleavage)	-
pKEHS1156	pET23	MH_6M-vtPA (Gly211-Pro562)	-
pFH249	pET23	MH_6M-vtPA (Gly211-Pro562)	DsbC
pKEHS1165	pET23	MH_6M-vtPA (Gly211-Pro562)	Erv1p
pFH219	pET23	MH_6M-vtPA (Gly211-Pro562)	Erv1p + DsbC
pVD122	pET23	Mature *E.coli* MBP (Lys27-Thr392) plus a linker GSGSGSGSGSIEGRGSGSGSGSGSHM - vtPA (Gly211-Pro562)	-
pVD171	pET23	Mature *E.coli* MBP (Lys27-Thr392) plus a linker GSGSGSGSGSIEGRGSGSGSGSGSHM - vtPA (Gly211-Pro562)	DsbC
pVD170	pET23	Mature *E.coli* MBP (Lys27-Thr392) plus a linker GSGSGSGSGSIEGRGSGSGSGSGSHM - vtPA (Gly211-Pro562)	PDI
pVD163	pET23	Mature *E.coli* MBP (Lys27-Thr392) plus a linker GSGSGSGSGSIEGRGSGSGSGSGSHM - vtPA (Gly211-Pro562)	Erv1p
pVD164	pET23	Mature *E.coli* MBP (Lys27-Thr392) plus a linker GSGSGSGSGSIEGRGSGSGSGSGSHM - vtPA (Gly211-Pro562)	Erv1p + DsbC
pVD165	pET23	Mature *E.coli* MBP (Lys27-Thr392) plus a linker GSGSGSGSGSIEGRGSGSGSGSGSHM - vtPA (Gly211-Pro562)	Erv1p + PDI
pVD103	pET23	Mature *E.coli* MBP (Lys27-Thr392) plus a linker NSSSNNNNHM - vtPA (Gly211-Pro562)	-
pVD105	pET23	Mature *E.coli* MBP (Lys27-Thr392) plus a linker NSSSNNNNHM - vtPA (Gly211-Pro562)	DsbC
pVD104	pET23	Mature *E.coli* MBP (Lys27-Thr392) plus a linker NSSSNNNNHM - vtPA (Gly211-Pro562)	PDI
pVD102	pET23	Mature *E.coli* MBP (Lys27-Thr392) plus a	Erv1p + DsbC

		linker NSSSNNNNHM - vtPA (Gly211-Pro562)	
pVD101	pET23	Mature *E.coli* MBP (Lys27-Thr392) plus a linker NSSSNNNNHM - vtPA (Gly211-Pro562)	Erv1p + PDI
pVD121	pET23	Mature *E.coli* MBP (Lys27-Thr392) plus a linker GSGSGSGSGSDDDDKHM - vtPA (Gly211-Pro562)	-
pHEE12	pET23	MH_6M-mature BPTI (Arg36-Ala93)	-
pHEE8	pET23	MH_6M-mature BPTI C49A / C73A (Arg36-Ala93)	-
pHEE9	pET23	MH_6M-mature BPTI C49A / C90A (Arg36-Ala93)	
pHEE11	pET23	MH_6M-mature BPTI C65A / C86A (Arg36-Ala93)	-
pHEE10	pET23	MH_6M-mature BPTI C73A / C90A (Arg36-Ala93)	-
pVD131	pET23	MH_6M-mature BPTI (Arg36-Ala93)	PDI
pOLR136	pLysS	Nothing extra. NsiI site added at 3071	-
pOLR137	pLysS	Nothing extra. AvrII site added at 3578 and NsiI site at 3071	-
pKEHS1205	pBAD102D-TOPO	AvrII site added at 1089 a XbaI site added at 316 and a XhoI site added at 796	-
pFH257	pLysS	*S.cerevisiae* Erv1p (Met 1 - Glu 189)	-
pFH256	pLysS	*S.cerevisiae* Erv1p (Met 1 - Glu 189)	PDI
pFH255	pLysS	*S.cerevisiae* Erv1p (Met 1 - Glu 189)	DsbC
pGZ9	pET23	MH_6M-mature human resistin (Lys19-Pro108)	-
pGZ16	pET23	MBPx- mature human resistin (Lys19-Pro108)	-
pKEHS1084	pET23	MH_6M-mature human Ero1α (Glu24-His468)	-
pKEHS1152	pET23	MH_6M-mature human CSF3 (Ala30-Pro207)	-
pKEHS1136	pET23	MH_6M-mature human CSF3 (Ala30-Pro207)	Erv1p
pVD196	pET23	MBP- mature human CSF3 (Ala30-Pro207)	Erv1p and DsbC
pVD197	pET23	MBP- mature human CSF3 (Ala30-Pro207)	Erv1p and PDI
pVD111	pET23	MBP- mature human CSF3 (Ala30-Pro207)	Erv1p and PDI
pVD108	pET23	MBPx- mature human CSF3 (Ala30-Pro207)	-
pKEHS1160	pET23	MH_6M- mature human BMP4 (Pro294-Arg408)	-
pVD142	pET23	MH_6M- mature human BMP4 (Pro294-Arg408) Cys372Ala	-
pKEHS1139	pET23	MH_6M- mature human BMP4 (Pro294-Arg408)	Erv1p
pKEHS1198	pET23	MH_6M- mature human BMP4 (Pro294-Arg408)	Erv1p and PDI
pKEHS1194	pET23	MH_6M- mature human BMP4 (Pro294-Arg408)	Erv1p and DsbC
pVD107	pET23	MBPx-mature human BMP4 (Pro294-Arg408)	-
pVD115	pET23	MBPx-mature human BMP4 (Pro294-Arg408)	Erv1p and DsbC
pVD129	pET23	MH_6M-light chain of human enterokinase (Ile785-His1019)	-
pVD130	pET23	MH_6M-light chain of human enterokinase (Ile785-His1019) Cys896Ser	-
pVD123	pET23	MBPx-light chain of human enterokinase (Ile785-His1019)	-
pVD124	pET23	MBPx-light chain of human enterokinase	-

		(Ile785-His1019) Cys896Ser	
pGZ10	pET23	MH_6M- mature human interferon α2 (Cys24-Glu188)	
pGZ15	pET23	MBPx- mature human interferon α2 (Cys24-Glu188)	
pMHR5	pET23	MBPx-mature interleukin 17 (Gly24-Ala155)	

The co-expressed proteins are either *S.cerevisiae* Erv1p (Met 1-Glu189); the mature form of *E.coli* DsbC (Asp21-Lys236); and/or the mature form of human PDI (Asp18-Leu508). Note that BPTI is listed here as for the full length protein while the numbering in the main text is for the mature protein, the difference between the two being 35 amino acids. Expression from pET23 plasmids is by IPTG induction, expression from modified pLysS plasmids is by arabinose induction. All pET23 vectors have a SpeI site added to the multi-cloning site.
